# Molecular Dynamics Simulation of Fatty Acid Extraction Using a Type V Deep Eutectic Solvent with Tunable Hydrophobicity

**DOI:** 10.1021/acs.jpcb.4c08261

**Published:** 2025-08-18

**Authors:** Petteri A. Vainikka, Matthijs J. Tadema, Siewert J. Marrink

**Affiliations:** † Zernike Institute for Advanced Materials, University of Groningen, Nijenborgh 4, 9747 AG Groningen, The Netherlands; ‡ Groningen Biomolecular Sciences and Biotechnology Institute, University of Groningen, Nijenborgh 7, 9747 AG Groningen, The Netherlands; § Centre for Analysis and Synthesis, Department of Chemistry, 5193 Lund University, Box 124, 221 00 Lund, Sweden

## Abstract

Deep eutectic solvents (DESs) are often promoted as a more environmentally friendly alternative for ionic liquids and other ionic solvents. Like ionic liquids, DESs can be designed for specific tasks in various chemical environments, but their usage in extraction processes is often significantly hampered by difficulties in recovering the extraction product. To remedy this, various tactics are being employed, which often decrease or completely remove the recyclability of the solvent. An experimental study from 2021 proposed a strategy to counter this issue, demonstrating how a hexanoic acid—imidazole (IMID) DES with “tunable hydrophobicity” can be used to extract fatty acids. This protocol did not involve the introduction of additional chemical species, thus better preserving the recyclability of the solvent. In this work, we reproduce the experimentally reported fatty acid extraction and recovery process using the coarse-grained Martini 3 force field. We demonstrate that our HexA-IMID DES simulations are capable of accurately reproducing the phase separation and extraction phenomena and yielding molecular level insights into the process. Additionally, we studied the effect of IL formation on these properties. Our study opens the way to rational design of new extraction protocols with DESs and further establishes the potential role CG MD can have in the screening of new DESs and their properties with comparatively little computational cost. Finally, we demonstrate that when combined with the Martini 3 IL models, we can efficiently study how different extents of reaction toward IL formation affect the system properties, as well as extraction properties.

## Graphical abstract

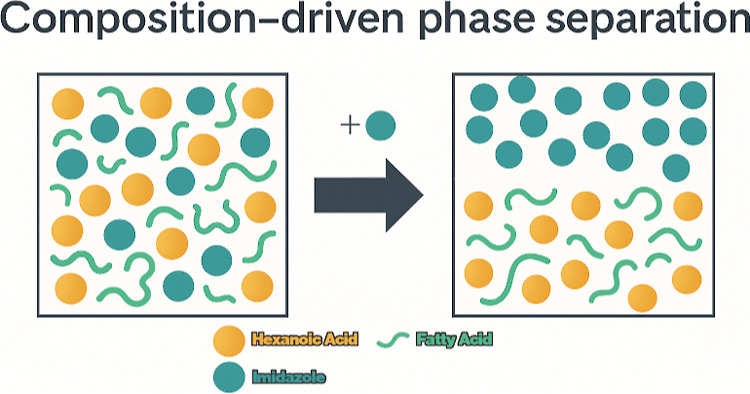

## Introduction

Deep eutectic solvents (DESs) are binary or ternary mixtures comprised of hydrogen bond acceptors (HBA) and hydrogen bond donors (HBD), which, when mixed have negative deviation from ideal behavior due to enthalpic effects.^1–3^ There is some controversy regarding how to classify a mixture as “DES”, since eutectic points are ubiquitous in mixtures, and there is no consensus on a global and transferable descriptor that would allow for an accurate classification of a DES. Despite the ambiguity regarding the definition, DESs themselves are clearly categorized into five different types. Types I–III consist of a quaternary ammonium salt with metal chloride, metal chloride hydrate, or a HBD, respectively. Type IV DES consists of a mixture between a metal chloride hydrate and a HBD.^4,5^ Type V DESs are the most recent class, being proposed by Abranches et al. in 2019.^6^ They consist of nonionic compounds, such as thymol or menthol.^7^

Most DESs are being synthesized and used in academic laboratory settings, and there are only a few fields of industry in which DESs have entered the exploratory phase of industrial use. A recent review by Yu et al. lists electrodeposition and eutectogel-based energy-storing DESs as the most promising candidates for industrial-scale use.^8^ One key issue in adopting DESs to industrial-scale processes has to do with viscosity: DESs are formed through the formation of an extensive hydrogen bonding network, which appears to, at least partially, yield the solvent many of its coveted solvation properties. However, the same H-bonding significantly increases the viscosity of the solvent, which hampers the mass transfer ratios and reduces the efficiency of the solvent. In most cases, this issue can be circumvented by either increasing the operating temperature of the solvent^9^ or by adding water as a ternary component. Unfortunately, both of these solutions create new problems, as increasing the temperature results in significantly higher energy consumption, and sufficient addition of water breaks the H-bond network, destabilizing the DES.^10,11^

Outside of experimental work, the computational and theoretical studies of DESs are primarily limited to quantum chemical calculations, COSMO-based models such as COSMO-RS and COSMO-SAC, and all-atom (AA) molecular dynamics simulations.^12–17^

While simulations at the AA resolution are powerful for analyzing fine-grained structural details, their high computational cost often limits them to system sizes and time scales that are insufficient for capturing large-scale phenomena. A coarse-grained (CG) approach, in which degrees of freedom are reduced by unifying 2 to 4 heavy atoms and their associated hydrogens into a single interaction site—or a bead, as they are referred to—offers a complementary approach. By sacrificing atomic detail, CG simulations can access the larger length and time scales necessary to study emergent processes like liquid–liquid phase separation and extraction with a reasonably high degree of accuracy.^18,19^ In our previous work we have demonstrated that CG molecular dynamics, based on the Martini model, can be applied to the study of DESs and other liquid–liquid phase separating systems as well.^20–22^ The ability of this approach to implicitly capture hydrogen bonding effects and reproduce correct structural features for DESs has been validated against both all-atom simulations and experimental data in our prior work.^20^

In this work, we further demonstrate the applicability of CG DES models by studying a potential type V DES, comprised of the HBD hexanoic acid (HexA), and the HBA imidazole (IMID), with the latest version of the Martini force field.^23^ Lo et al. have shown that the hydrophobicity of this solvent can be “tuned” by modifying the ratio between the two constituent components.^24^ When the mole fraction of IMID is increased from the initial value of χ_imid_ = 0.30 to approximately χ_imid_ = 0.60, the solvent phase separates to HexA-rich and IMID-water-rich phases. This was subsequently harnessed to perform extractions: Lo and colleagues managed to extract fatty acids from sunflower oil and algae oil, which partition into the HexA phase. The original mixed state can then be recovered by adding more HexA, potentially allowing for higher recyclability. Adding to the previous work, we attempt to quantify the possible effects ionic liquid (IL) formation has on the extraction properties, as HexA and IMID potentially could react to form imidazolium (IMID+) hexanoate (HexA-).^25^

## Methods

### Computational Details

All simulations were performed with GROMACS 2021.3 ^26,27^ using the Bussi-Donadio-Parrinello thermostat (V-rescale)^28^ with a time constant of 1.0 ps. Simulation pressures were controlled with the Berendsen barostat^29^ during equilibration and with the Parrinello–Rahman barostat^30^ during production simulations (with a time constant of 12.0 ps and a compressibility of 3 × 10^–4^ bar^–1^). Constraints, where applied, were solved using LINCS,^31^ with a LINCS order of 4. Electrostatic interactions were computed by using the reaction-field method. Viscosity was calculated using the periodic perturbation method.^32^ Finite-size effects were accounted for by systematically increasing the system sizes and extrapolating to an infinite system size.

### Martini 3 CG Models

Components used in this work included HexA, hexanoate (HexA-), IMID, imidazolium (IMID+), oleic acid, linoleic acid, and water. Water is CG as a single bead, which represents four water molecules. IMID has been previously published as a part of the Martini 3 small molecules library,^33^ from which the bonded potentials for the imidazolium model were adapted. Intermolecular parameters were derived from previously published Martini 3 imidazolium models.^22^ HexA, hexanoate, oleic acid, and linoleic acid parameters were derived based on the existing Martini 3 fatty acid and carboxylic acid models, either distributed with the original Martini 3 publication^23^ or with the Martini DES publication.^20^ All CG models are illustrated in Figure 1. Details on the validation of the models are given in Supporting Information, Sections S1 and S2.

**Figure 1:**
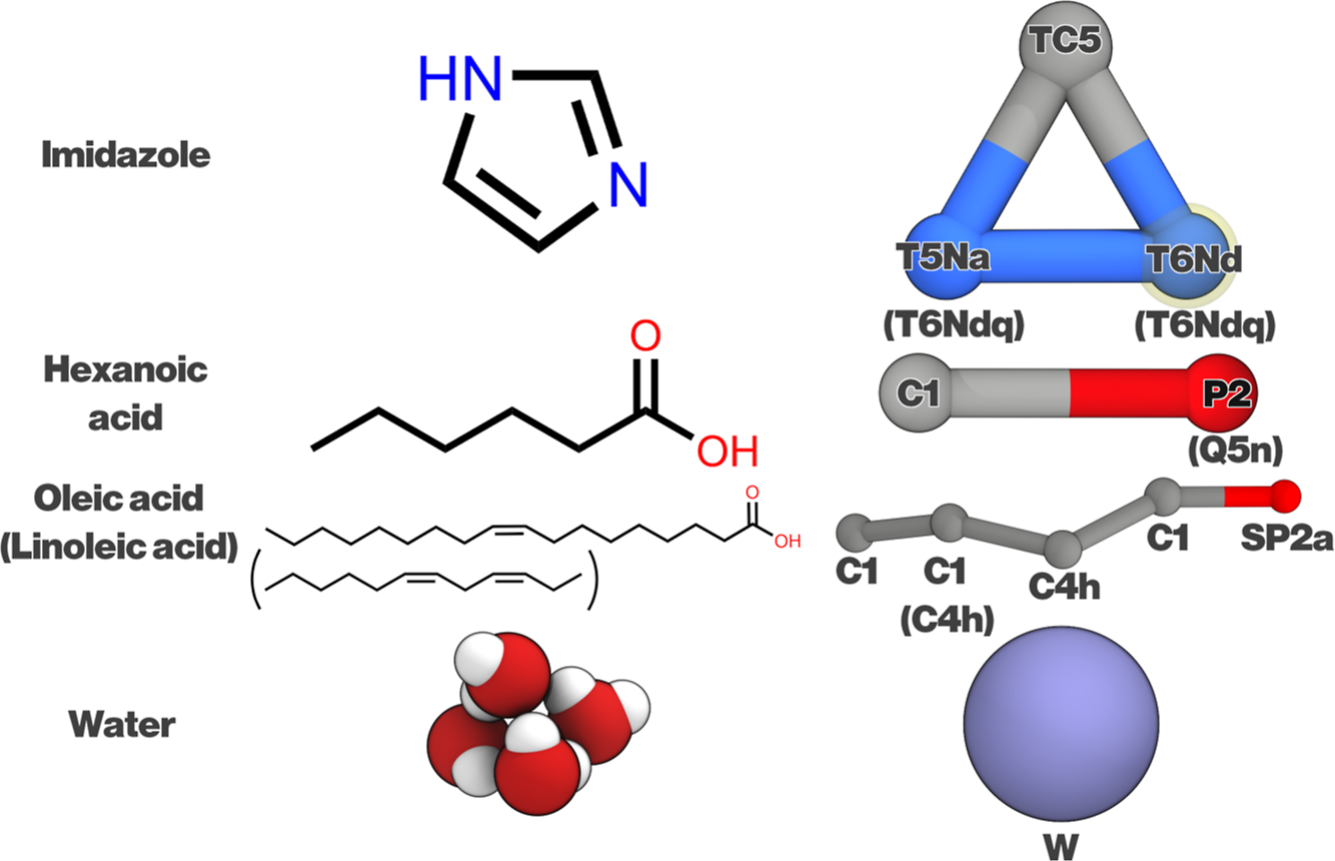
Components used in this study. CG oleic acid and linoleic acid differ by one bead type, as indicated by the parentheses. Alternative bead assignments are used as IMID and HexA react to form an [HexA]^−^[IMID]^+^ IL. These are given in parentheses below their neutral counterparts. Water, as is the standard in Martini 3, is represented by a specific water bead (“W”) and is comprised of four individual water molecules.

### Simulation Setups

Starting configurations were generated randomly with gmx insert-molecules. Every system was subjected to the same minimization and equilibration routine: minimization with the steepest-descent algorithm, followed by a 100 ps long initial equilibration step with 1 fs time step and 200 K temperature, followed by a 500 ps equilibration step with 2 fs time step at 290 K, and a final 50 ns equilibration step at target temperature (in the case of temperature-dependent properties) or at 290 K with a time step of 20 fs. Equilibration was confirmed for all systems by monitoring properties, such as the potential energy and density, to ensure they had reached a stable plateau.

The mole fraction of water was set to 0.1 for each system in this study. We chose this value based on the knowledge that properties of most DESs, such as viscosity, change dramatically as small amounts of water are added to a completely dry DES. On the other hand, the change induced by adding more water to already hydrated DESs is significantly less pronounced.^34,35^ The original study did not report the amount of water present in their systems.

### Thermodynamic and Kinetic Properties

Diffusion coefficients and thermal expansion coefficients were all derived from the same set of simulations. Diffusion coefficients were calculated using the Einstein relation according to eq 1, and thermal expansion coefficients were obtained from bulk densities according to eq 2.

1 $$\lim_{t\to \infty }{\left\langle {\left|\left|r_{i}\left(t\right)-r_{i}\left(0\right)\right|\right|}^{2}\right\rangle }_{i\in A}=6D_{A}t$$

2 $$\alpha =-{\left(\frac{\partial \ln\rho }{\partial T}\right)}_{p}$$

We used 7 different compositions (χ_IMID_ = 0.10, 0.20, 0.25, 0.30, 0.35, 0.40, 0.50), which were each simulated in a temperature range from 270 to 330 K, with 10 *K* spacing. For each temperature, we created 3 system sizes (10^4^, 20^4^, and 30^4^ beads), and each size was simulated with 4 separate replicas for 300 ns, totaling 588 simulations and 176.4 μ s of combined simulation time. Finite size effects of diffusion were corrected for by plotting the measured values as a function of *N* ^–1/3^, where N is the number of particles present in the simulation, and extrapolating to an infinite-sized system. Average standard errors for the diffusion coefficients were obtained by averaging the variance of the error estimates for each individual measurement. Standard deviations for densities and thermal expansion coefficients were obtained as averages over various system sizes while keeping the system composition and temperature constant. The standard error of the mean of viscosity was calculated from error estimates given by gmx energy.

The extent of the phase separation was quantified by measuring the number of contacts between components in each simulation using the tool gmx mindist and normalizing it in relation to the overall number of contacts, as is described in eq 3.

3 $$100\%\times \frac{N_{contacts}\left(HexA-IMID\right)}{N_{contacts}\left(HexA-IMID\right)+N_{contacts}\left(IMID-IMID\right)}$$

here, *N*_contacts_ represents the number of molecular pairs within a cutoff distance of 0.6 nm, as calculated by the gmx mindist tool. This metric provides a quantitative measure of phase separation by relating the number of contacts between dissimilar components (HexA-IMID) to the total contacts involving IMID. In a well-mixed system, this value is high, while in a phase-separated system, where IMID molecules preferentially self-associate, the percentage of contacts with HexA decreases significantly. The resulting percentage was then related to the miscibility of individual bead types of Martini 3, which are given in the Supporting Information of the Martini 3 publication^23^ (Supporting Information, page 18, Table 20). The three relevant classifications were “mixed” (>50%), “partially mixed” (30–45%), or “almost biphasic” (15–30%).

Viscosity was estimated using the periodic perturbation method^32^ using a similar set of simulations as before, with the same composition and temperature ranges. Within each temperature, we used 7 acceleration rates, ranging from 0.002 nm/ps^2^ to 0.008 nm/ps^2^, with 0.001 nm/ps^2^ spacing. For each acceleration rate, we simulated 4 replicas, totaling to 1372 simulations. Each system was built such that the *z*-axis was elongated to be 3 times as large as the *x*/*y*-axis. The systems were minimized and equilibrated following the previously discussed routine and then simulated for 3 ns with a 5 fs time step.

### Per-Phase Diffusion Analysis

Per-phase diffusion coefficients were calculated for a representative subset of systems (χ_IMID_ = 0.10, 0.30, and 0.50 at 270, 300, and 330 K). Molecules were classified into HexA-rich and IMID-rich phases based on their local molecular composition within a 1.2 nm radius. To avoid survivor bias (i.e., selectively measuring only slow-moving molecules that remain in a phase), the mean squared displacement (MSD) was calculated for molecules grouped by their phase of origin. Final diffusion coefficients were derived from the linear regime of the MSD curves and corrected for finite-size effects via extrapolation to an infinite box size.

### IL Formation

Effects of reaction toward imidazolium hexanoate were studied, both in order to understand what structural changes occur and to investigate how the presence of the IL affects the extraction properties. This was achieved by increasing the extent of the DES → IL reaction in a stepwise manner; 25%, 50%, 75%, and finally all of IMID was changed to IMID+, with a corresponding amount of HexA being changed to HexA-. As each system studied in this work contains some water (i.e., $\chi_{H_{2}O}=0.1$ ), the systems in which χ_IMID_ ≥ 0.45 have more IMID than HexA, leaving some of the IMID unreacted.

### Extraction Properties

To directly investigate the experimentally observed phase separation, the extraction of fatty acids was studied using a composition range of χ_IMID_ = 0.30 to 0.60, which mirrors the critical range reported by Lo et al., in the original experimental work. All systems were built by randomly placing molecules within the simulation volume. Preproduction steps were performed as described previously, and the production simulations were run for 1 μ s. The extent of the phase separation was quantified by studying the radial distribution functions (RDFs) and partial density profiles of each component as a function of χ_IMID_. In order to quantify the effect IL formation has on the extraction, we performed the same set of simulations with two extents of reaction; one set had half of all HexA reacted with IMID to form imidazolium hexanoate, and the other set had full extent of reaction.

## Results

### Thermodynamic and Kinetic Properties

After visually inspecting the simulations at varying χ_IMID_ and temperatures, it became obvious that the systems are able to capture the phase separation as described in the original study by Lo et al.^24^ As χ_IMID_ is increased to 0.30, IMID can be visually observed aggregating. At higher values of χ_IMID_, the phase separation becomes more clear, as can be seen in Figure 2.

**Figure 2:**
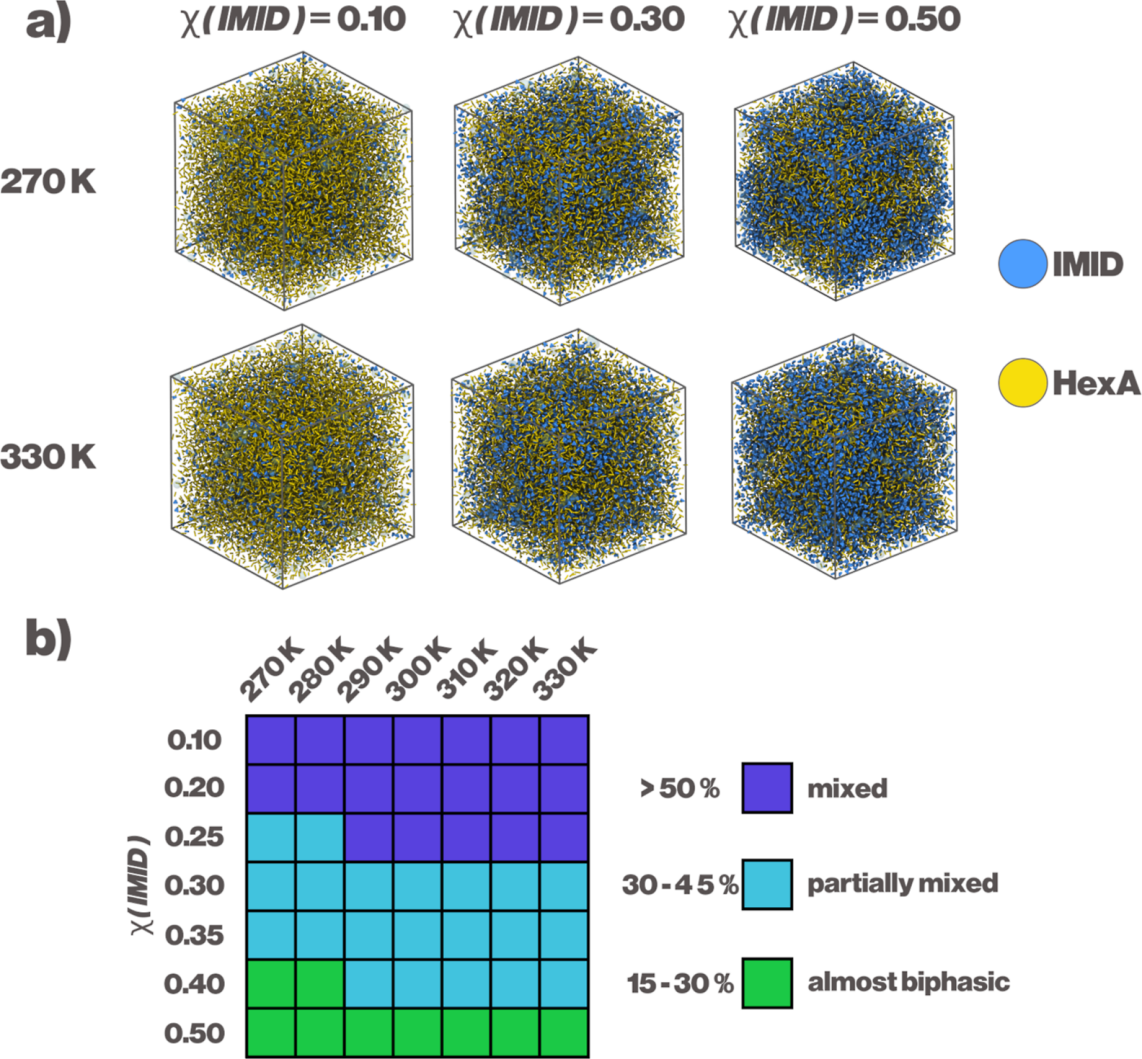
Miscibility of IMID and HexA. (a) Snapshots from the simulations at varying IMID content, at 270 and 330 K. (b) Miscibility table of the DES based on number of contacts. More than 50% contacts between IMID and HexA is considered a well-mixed state, 30–45% contacts partially mixed, and less than 30% contacts is considered almost biphasic. Percentages are calculated according to eq 3.

A more quantitative approach, based on the percentage of the number of contacts between the individual components in the simulation, indicates three distinct types of mixing behavior, as shown in the same figure. The numerical data is given in the Supporting Information, Section S3.

In the experimental study replicated in this work, viscosities, bulk densities (or thermal expansion coefficients), diffusion properties, effects, and quantities of water present in the solvent were not quantified. Despite the lack of experimental data, we decided to scan the thermodynamic and kinetic properties of the HexA-IMID DES within the “operating composition range” reported in the original study.^24^ Densities, shear viscosities, and diffusion coefficients were studied within the range χ_IMID_ = 0.1–0.5, with the hopes of combining, and potentially correcting, our models and data with further experimental data in the future.

Bulk densities and thermal expansion coefficients (α) of the HexA-IMID DES, with varying amounts of IMID, are presented as a function of temperature in Figure 3. The density profiles are as expected, decreasing as a function of temperature and increasing as the mole fraction of the H-bond accepting IMID increases. However, the thermal expansion coefficient indicates a more subtle change: values of α increase linearly up to 0.25, after which they level off, possibly indicating the transition from a solvent with bulk HexA properties to a HexA-IMID DES.

**Figure 3:**
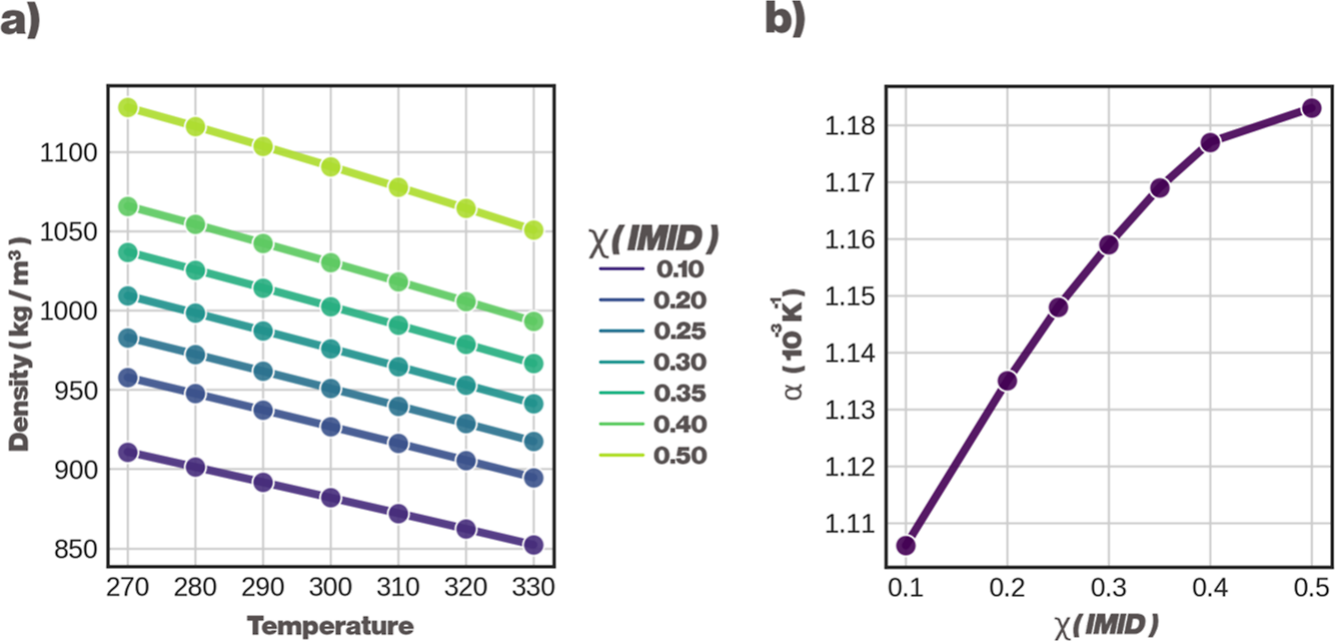
Thermodynamic properties of a HexA-IMID DES. (a) Density as a function of temperature, with varying χ_IMID_ and (b) thermal expansion coefficient (α) as a function of χ_IMID_. Error estimates are smaller than the size of the dots representing the data points in all cases.

This is also reflected in the measured diffusion coefficients, which are illustrated for both major components (HexA and IMID) in Figure 4. In general, the behavior is as expected, with the diffusion coefficients increasing as the temperature is increased. HexA shows the largest deviations from linear response within the range χ_IMID_ = 0.25–0.30, albeit more strongly at higher temperatures. IMID has similar deviations, especially at higher temperatures, at χ_IMID_ = 0.10—due to the small amount of IMID molecules present—but more deviations from linear behavior can be seen at χ_IMID_ = 0.30, indicating that the system is undergoing a possible transition toward forming a DES. It should be noted that the dynamic properties calculated from CG models, such as diffusion and viscosity, are known to deviate from experimental values due to the inherently smoothed free-energy landscape. For this reason, our conclusions are not drawn from the absolute quantitative values but from the robust qualitative trends observed in our simulations. These trends, such as the nonlinear behavior around χ_IMID_ = 0.30, are the key indicators of the underlying structural transitions within the solvent.

**Figure 4:**
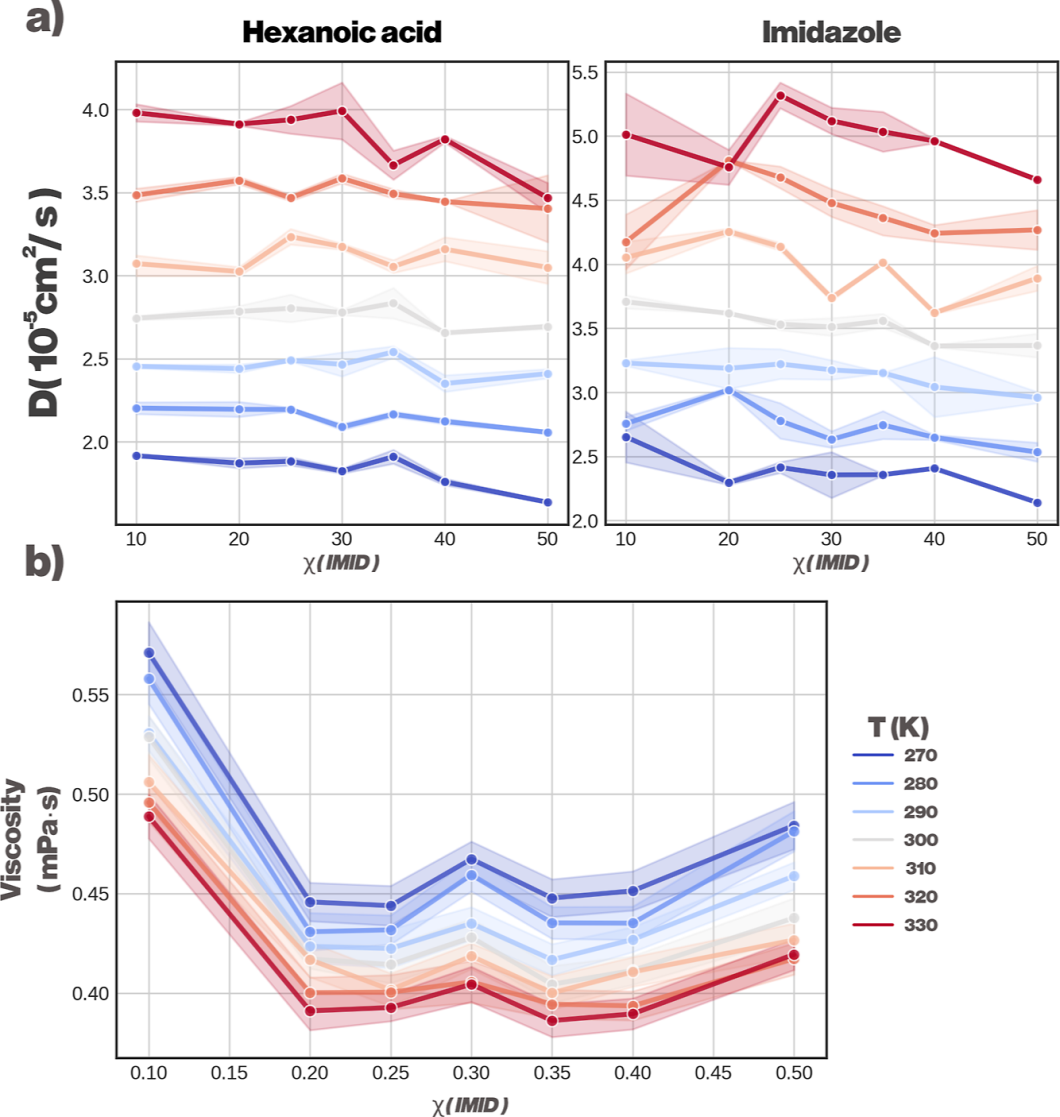
Kinetic properties of a HexA-IMID DES. (a) Diffusion coefficients of HexA (left) and IMID (right) a function of temperature, with varying χ_IMID_. (b) Shear viscosity as a function of χ_IMID_. Shaded areas indicate the standard error of the mean.

To gain deeper insight into the dynamics of the system following the onset of nanoscale phase separation (χ_IMID_ ≥ 0.30), we performed a rigorous per-phase diffusion analysis. The complete, finite-size-corrected results for a representative subset of systems are provided in the Supporting Information (Table S6).

The results of this analysis are highly informative: for all compositions and temperatures where two phases were present, the calculated diffusion coefficient for a given molecular species was found to be statistically identical, regardless of whether the molecule originated in the HexA-rich or IMID-rich phase. This key finding quantitatively demonstrates that even when structurally heterogeneous, the system behaves as a single, dynamically homogeneous fluid. The rate of molecular exchange between the transient nanodomains is sufficiently rapid that the long-term mobility of a molecule is governed by the average properties of the entire system. This provides a strong data-driven justification for the use of bulk properties to characterize the overall kinetic behavior of this complex fluid.

Shear viscosities, estimated with the periodic perturbation method, are shown in Figure 4. The temperature dependency of the shear viscosity is as expected, with viscosity decreasing as temperature is increased. The behavior as a function of χ_IMID_, however, is more interesting. Based on the density profiles, one would expect an upward trend in viscosity as χ_IMID_ is increased, yet we notice a sharp decline in the shear viscosity. This is likely due to perturbation of HexA–HexA and HexA-water interactions, induced by increased amounts of IMID. At χ_IMID_ = 0.30, we observe a peak, likely corresponding to the aforementioned formation of the DES. The shear viscosity values then steadily increase as more and more IMID is added, reflecting the strong self-interaction of IMID, which is both a HBA and a HBD, taking over the dynamics of the system.

### Extraction Properties

The original study^24^ used the HexA-IMID DES to extract fatty acids from sunflower oil and algae oil. To capture the extraction process in our simulations, algae oil was modeled as pure oleic acid (OLEC), as it is >90% oleic acid by mass. Sunflower oil has two primary constituents, oleic acid (approximately 30%) and linoleic acid (approximately 60%), and was modeled as pure linoleic acid (LINO) in order to study the difference between extracting a monounsaturated fatty acid and a polyunsaturated one.

Simulations started from a completely mixed state, with the system containing HexA, IMID, water, and either OLEC or LINO. The initial simulations contained HexA and IMID in a 2:1 ratio, with $\chi_{H_{2}O}=0.1$ and 0.28 g oil/g solvent, as was the case in the experimental setup. The mole fraction of IMID was then increased, while the amount of other compounds was kept constant.

Experimentally,^24^ the phase diagram shows separation of the DES/oil mixture into a HexA phase enriched in oil and an IMID/water phase depleted of oil, to occur between χ_IMID_ = 0.40 and 0.50. This was immediately observed within the simulations by visual inspection; a comparison between a system with χ_IMID_ = 0.30 to a system with χ_IMID_ = 0.50 shows easily observable accumulation of IMID in its own domain, whereas the fatty acid and HexA remain separated. This is shown in Figure 5 with snapshots of both systems.

**Figure 5:**
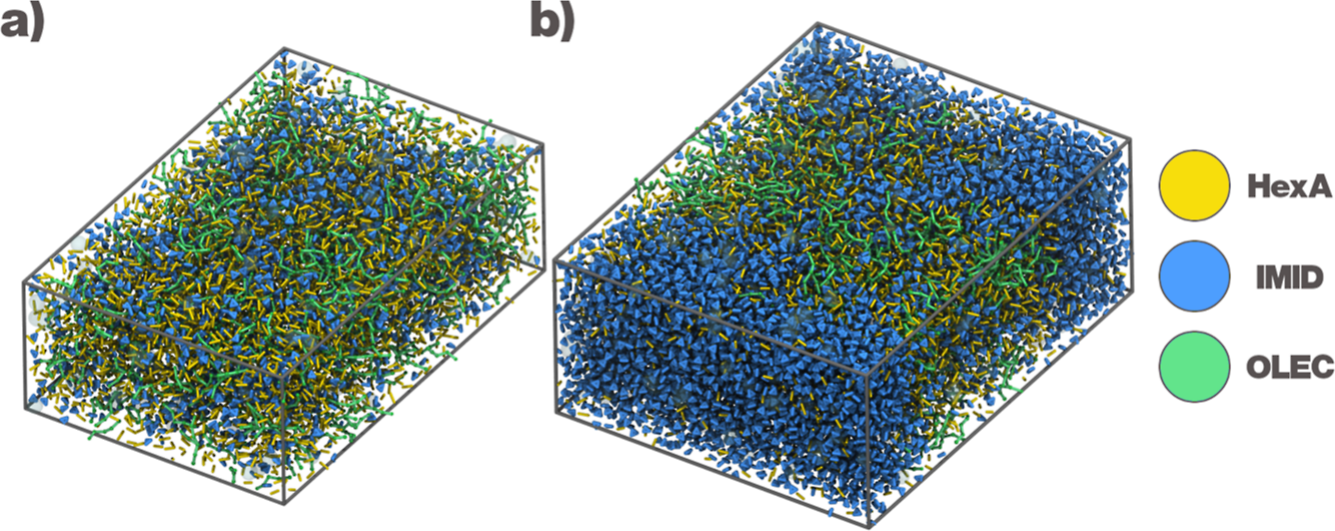
Phase separation in the HexA-IMID DES in the presence of oil. (a) A χ_IMID_ = 0.30 system, showing a mixed state with very small aggregates of IMID. (b) A χ_IMID_ = 0.50 system, with clear IMID-rich domains.

To obtain a more quantitative idea of the phase separation, we analyzed the partial densities of each component as a function of the mole fraction of the IMID. This is illustrated in Figure 6.

**Figure 6:**
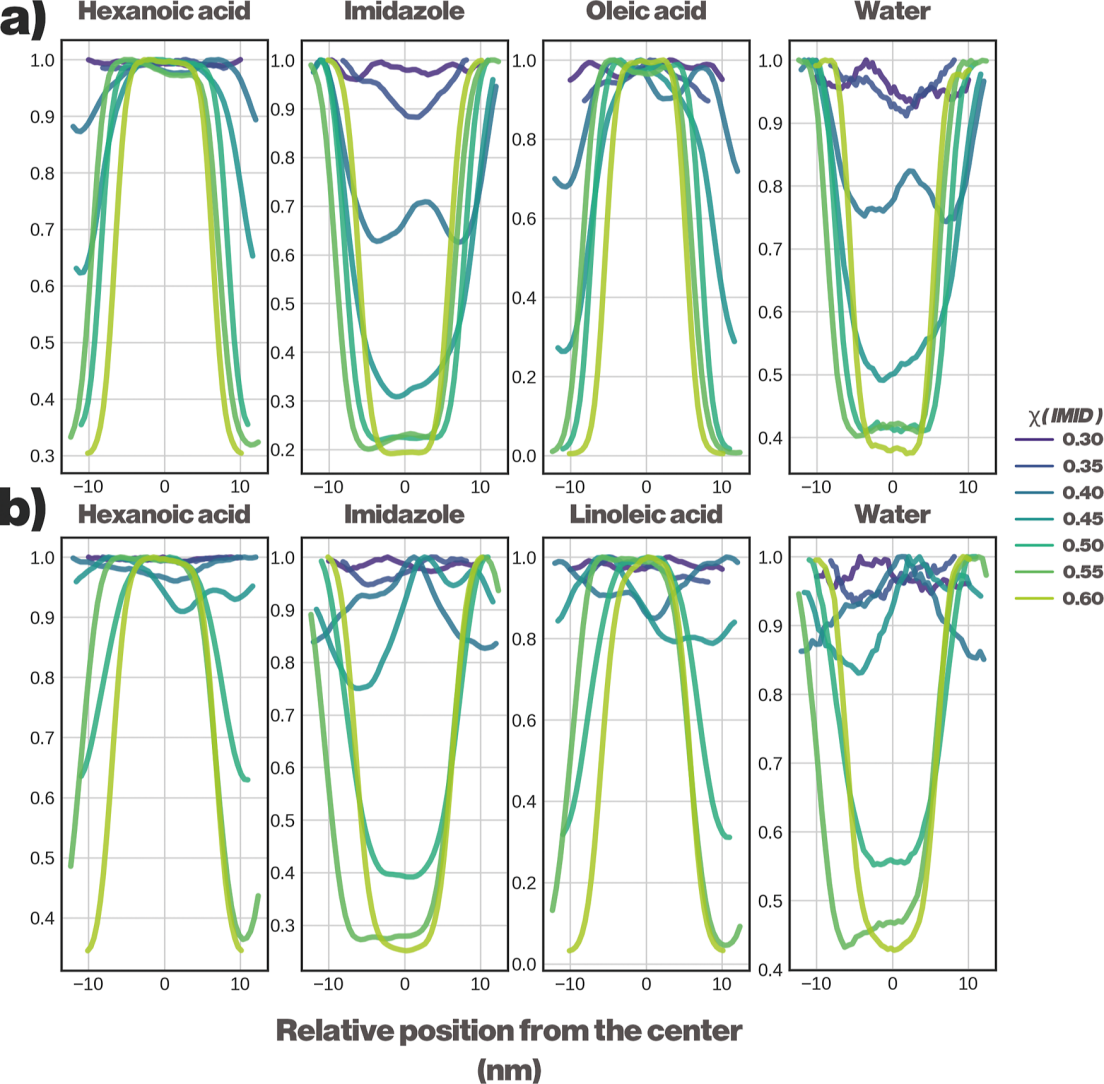
Spatial distribution of system components of a HexA-IMID DES in the presence of oil. (a) Normalized partial density profiles of DES components mixed with oleic acid. (b) Normalized partial density profiles of DES components mixed with linoleic acid.

Our simulations provide a clear molecular-level explanation for the tunable hydrophobicity reported in the experimental work. This phenomenon does not arise from a change in the intrinsic properties of the individual molecules but rather from a composition-driven phase separation of the entire solvent system. As the mole fraction of IMID is increased to values above χ_IMID_ = 0.40, the polar IMID and water molecules begin to self-aggregate, forming distinct, IMID-rich domains. This process, visualized in Figure 5 and quantified by the partial density profiles in Figure 6, leads to the formation of a discrete polar phase. The creation of this polar environment effectively expels the less polar HexA and the dissolved fatty acid, forcing them to partition into their own colocated, nonpolar phase. Thus, the ability to switch the system from a homogeneous solvent to a biphasic liquid simply by altering the component ratio is the direct molecular origin of the tunable hydrophobicity that facilitates the extraction process.

At the lowest amounts of IMID, we can see the system remaining in a mixed state, as indicated by the flat purple lines in Figure 6. As more IMID is added, the system can be observed responding by forming IMID-water-rich domains and eventually separating to OLEC-HexA and IMID-water phases. The original study noted that the fatty acid phases still contain some amount of IMID as a pollutant; the simulations concur with the statement. Approximately 15 to 20% of the IMID still remains outside of the IMID-water phase. The same analysis of the simulations containing the polyunsaturated LINO indicates that the system undergoes phase separation as the OLEC-containing systems do, but the onset of the phase separation is observed only at higher values of χ_IMID_. This is most likely due to the higher affinity the polyunsaturated LINO has toward IMID. This also explains the higher amount of IMID (25–30 mol %) that remains as a pollutant within the HexA—LINO phase.

To further study the nanolevel structure present in the HexA-OLEC phase, we computed RDFs between all components present in the simulation. RDFs for the key pairs are shown in Figure 7. All RDF data are shown in the Supporting Information, Section S5.

**Figure 7:**
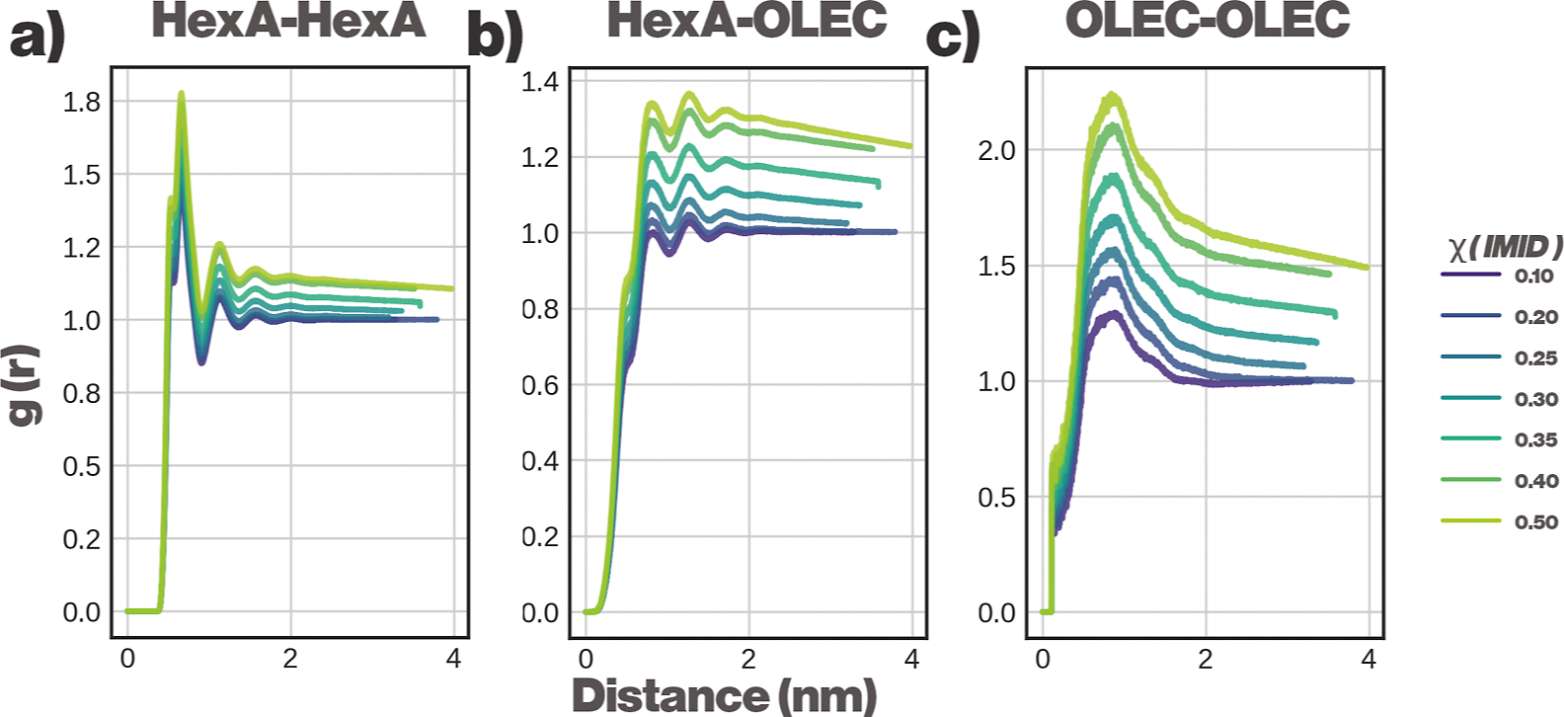
RDFs as a function of χ_IMID_. (a) HexA-HexA, (b) HexA-OLEC, and (c) OLEC-OLEC pair-correlation. Although HexA and OLEC separate to the same phase, the RDF profiles indicate that both compounds have a preferential self-interaction over the HexA-OLEC interaction.

Figure 7 shows that the first solvation shell of OLEC is preferentially occupied by other OLEC molecules, and the first solvation shell of HexA is preferentially occupied by other HexA molecules. Although this segregation is not strong enough to be observed visually from the simulations, it is possible that significantly larger systems could undergo further phase separation between HexA and OLEC. The driving force between the observed nanolevel segregation is likely the stabilizing interaction between longer aliphatic tails of OLEC.

### Probing the Effect of IL Formation

As has been shown earlier, it is likely that the two DES components react to form an IL. Yet, based on the limited amount of experimental evidence available, the extent of this reaction remains unclear. Intriguingly, our simulations suggest that the DES → IL-reaction is not likely to reach completion; the experimental study reported a clearly visible two-phase system where excess HexA and the fatty acid are separated from the IMID-rich phase. When the reaction is allowed to run to completion, we observe the pure IL separation from the fatty acid, but the phases are not as clearly separated as with the DES. These results are illustrated in Figure 8.

**Figure 8:**
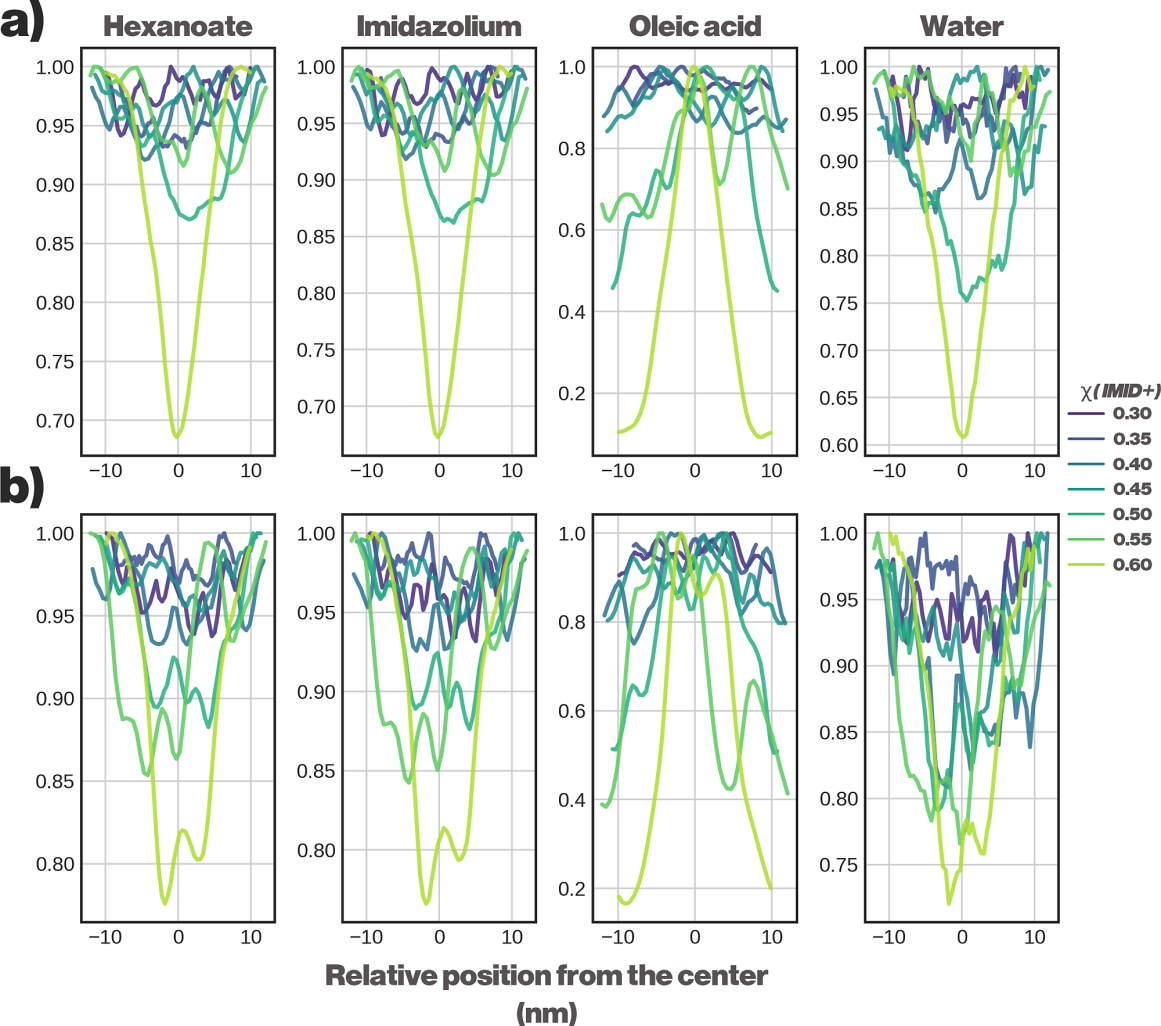
Spatial distribution of system components of a imidazolium hexanoate IL in the presence of oleic acid. (a) 50% extent of reaction, showing a limited separation between IL and oleic acid, with water partitioning within the IL. (b) 100% extent of reaction, showing a further diminished phase separation.

Further characterization of the effects of IL formation was performed by studying miscibility of constituent components. The models predict a strong phase separation at low χ_IMID_ values when the reaction is allowed to proceed to completion. Interestingly, at lower extents of reaction, the system remains in a mixed state, suggesting that the formed IL can readily solubilize IMID. Furthermore, when the extent of the reaction (ξ) reaches 75%, we observe the system moving from a partially mixed state to an almost biphasic state as the amount of IMID is increased, with even further addition of IMID reverting the system back to a partially mixed state. This is in line with the findings reported in Figure 8 in the main text, where only a weak phase separation is observed at ξ = 100%. The full miscibility data for ILs are given in the Supporting Information, Section S4.

### Solvent Regeneration

A key feature of the original study by Lo et al. was the protocol of inducing phase separation via the addition of IMID, which allows for energetically cheap recovery of the fatty acids. As the authors point out, the method is still not fully feasible, as the regeneration of the solvent is dependent on the addition of more HexA, eventually leading to a buildup of the solvent and possibly nullifying the environmental benefits gained. We examined if our models could be reverted back to the mixed state from the phase-separated state and quantified the amount of HexA required to do so. Taking the final configuration of one of the χ_IMID_ = 0.60 simulations, we kept adding HexA until we could visually observe a recovered mixed state. Snapshots of these simulations are presented in Figure 9. The mixed state was visually observed to have been regenerated after the addition of HexA in such quantities that χ_IMID_ = 0.30. Altogether, the mass of the solvent had to be increased by a factor of 2.4, demonstrating the challenge of sustaining the proposed extraction cycle.

**Figure 9:**
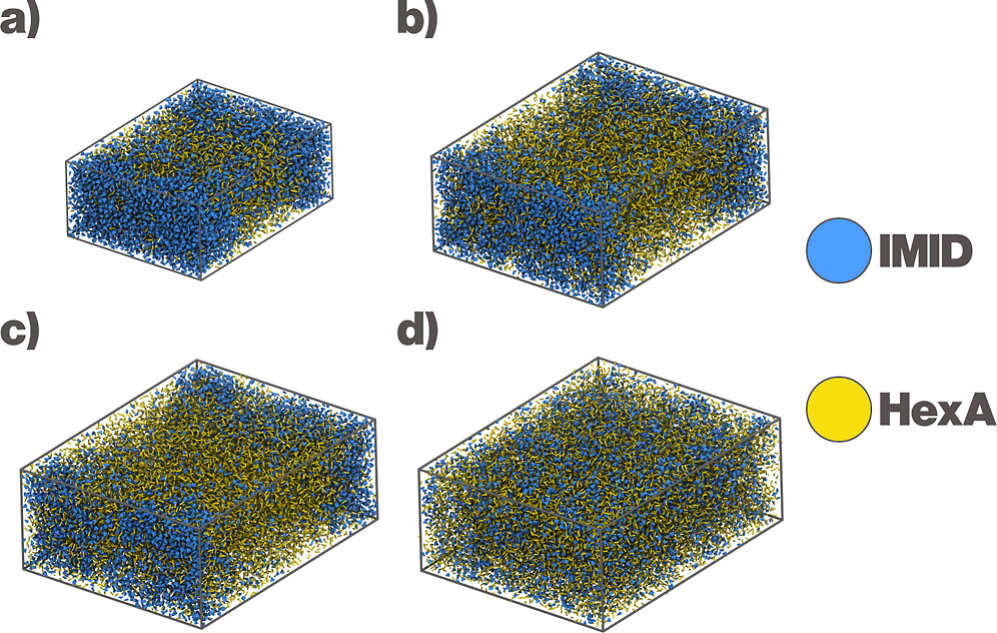
Regeneration of the HexA-IMID DES. (a) Phase-separated state, (b,c) addition of HexA, and (d) regenerated DES in a mixed state.

## Conclusions

We demonstrate the first use case for a Martini CG model of a type V DES composed of HexA and IMID. In addition to predicting the thermodynamic and kinetic properties of the solvent inside the composition range used in experimental measurements, we show that our models are able to capture the experimentally observed phase separation phenomena when mixed with oil and the preferential partitioning of fatty acids in the HexA phase. Investigation of the effect of the DES → IL reaction strongly suggests that although some IL is likely forming during the experiment, the reaction does not proceed to completion, as this would compromise the experimentally observed phase separation. This work indicates that Martini 3, and perhaps other similarly parametrized CG force fields, are a useful tool for studying the dynamics of DESs, and are promising in the study of processes such as liquid–liquid extraction. In the long term, we envision establishing CG MD as a method for identifying and characterizing dynamics and properties of possible novel DESs.

## Supplementary Material



## Notes

Details on model validation including partition coefficients and surface areas; characterization of the IL models; tabulated data for contact analysis and miscibility; full RDFs; and per-phase diffusion coefficients (PDF)
