# Supplementary material for: Molecular Dynamics Simulation of Fatty Acid Extraction Using a Type V Deep Eutectic Solvent with Tunable Hydrophobicity
Source: J Phys Chem B. 2025 Aug 18;129(34):8754–63. doi: 10.1021/acs.jpcb.4c08261 (PMC12400407; doi:10.1021/acs.jpcb.4c08261)
Supplement: Supplementary file 1 [file jp4c08261_si_001.pdf]

Molecular Dynamics Simulation of Fatty Acid Extraction Using a Type V Deep Eutectic Solvent with Tunable Hydrophobicity

Supporting Information

Petteri A. Vainikka[1,2,3], Matthijs J. Tadema[1], and Siewert J. Marrink[1,2]*

[1] Groningen Biomolecular Sciences and Biotechnology Institute, University of Groningen, Nijenborgh 7, 9747 AG Groningen, The Netherlands
[2] Zernike Institute for Advanced Materials, University of Groningen, Nijenborgh 7, 9747 AG Groningen, The Netherlands
[3] Centre for Analysis and Synthesis, Department of Chemistry, Lund University, Box 124, 221 00 Lund, Sweden

* Corresponding Author
Email: s.j.marrink@rug.nl (S.J.M).

Number of pages: 9
Number of figures: 4

Number of tables: 6

1. Partition coefficients and solvent accessible surface areas

As partitioning played a key role in this study, we estimated the partition coefficient (log P) for each model using the thermodynamic integration (TI) method, following the same protocol as with the previous CG DES models[1] and compared the results against experimental and other predicted values. Correctness of the bonded terms was tested by comparing the solvent accessible surface area (SASA) of the CG models against atomistic counterparts. We used a probe radius of 0.191 nm and 4800 dots per sphere for both resolutions. Analysis on the the resulting distribution was performed with *gmx analyze*, using a bin width of 0.01. Atomic radii used to evaluate the AA models were taken from Rowland *et al.*[2] Partition coefficients for these compounds, along with experimental and other predicted values, are presented in Table SI1. The SASAs are given in Table SI2.

Table S1: Log P values obtained with Martini 3, experimental values with their respective references, and predicted values with the software they were predicted with.

Compound	Martini 3	Experimental	Ref.	Predicted	Software
**Hexanoic acid**	1.85 ± 0.03	1.92	3	1.88	AlogPs
**Imidazole**	-0.38 ± 0.01	-0.08	3	0.10	XLogP3
**Oleic acid**	8.86 ± 0.12	7.64	4	7.68	AlogPs
**Linoleic acid**	7.59 ± 0.02	6.46	4	6.62	AlogPs

Table S2: Measured SASA values for each CG model created for this study, and its AA counterpart. Positive difference indicates the Martini model to be larger than the atomistic model, and vice versa.

Compound	Martini 3 (nm^2)	All atom (nm^2)	Difference (%)
**Hexanoic acid**	3.72 ± 0.07	3.79 ± 0.06	-1.8
**Imidazole**	2.86 ± 0.01	2.62 ± 0.01	9.1
**Oleic acid**	7.63 ± 0.18	7.24 ± 0.52	5.3
**Linoleic acid**	7.19 ± 0.37	7.04 ± 0.54	2.1

The partition coefficients of oleic- and linoleic acid are overestimated roughly by an order of magnitude, but both the experimental and the computationally derived values point to extremely hydrophobic behaviour, which we can safely assume to be captured based on the values derived from our models. SASA values are deemed acceptable if they are within 10% of the atomistic counterpart. The largest deviation (9.1 %) was observed in the case of the previously

parameterized imidazole model, which was still below the 10 % threshold. Taken together, we deemed the bonded and non-bonded terms to be of acceptable quality.

2. Brief validation of the ionic liquid models

In order to investigate the effects of ionic liquid formation within our system, we derived models from imidazolium and hexanoate from their corresponding neutral models. Derivation of the hexanoate model was straightforward, with a single bead being replaced (P2 →Q5n) and a negative unit charge placed on the conjugate base. The model for imidazolium was based on the previous work on Martini 3 ionic liquids[5], which already contain models for various imidazolium cations, including a structurally similar 1-methylimidazolium, which was used as a reference when considering partial charge placement.

As the main focus of the paper was in the behaviour of the deep eutectic solvent, and as the required modifications to the pre-established IL models were extremely limited, we chose only to perform minute testing of the models. This testing involved using the pure IL and comparing its predicted structural characteristics against an all-atom model of the same IL, and matching a limited number of reported experimental values from literature. Matching of structural characteristics between resolutions is illustrated in Figure SI1 below.

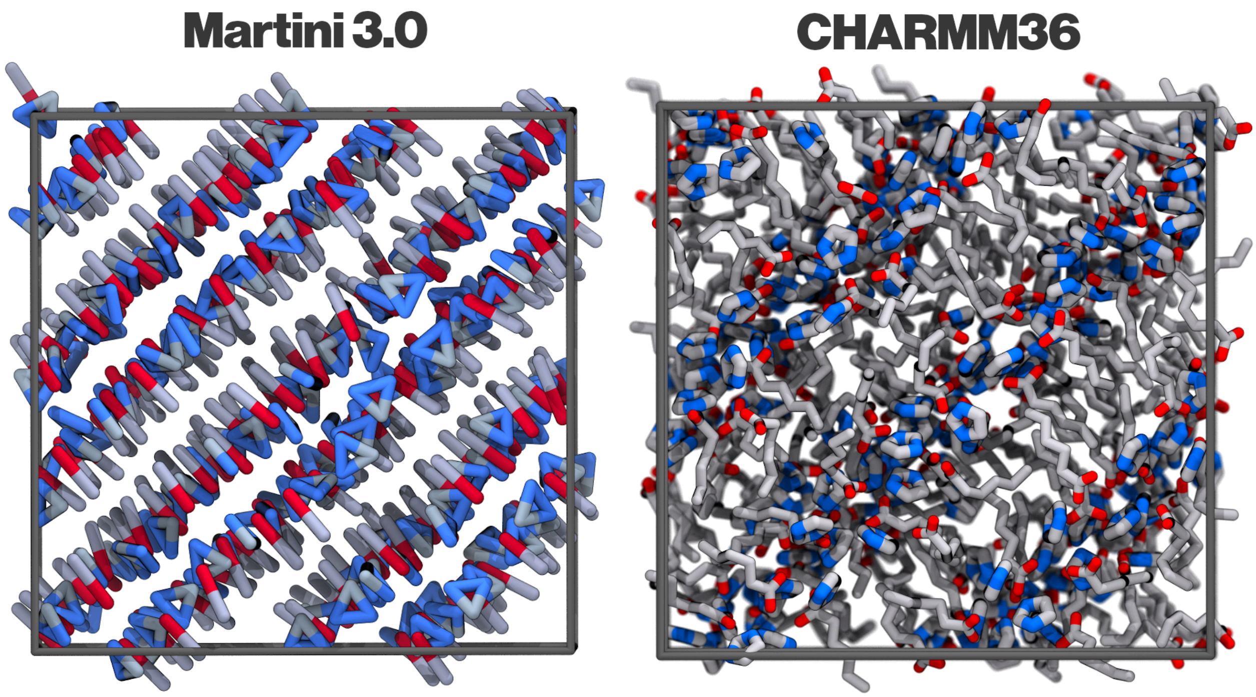

Figure S1: Lamellar structure of imidazolium hexanoate, as predicted by Martini 3.0, and CHARMM 36.

We found that the lamellar structure predicted by Martini 3.0 was fully in line with the structure predicted by CHARMM36. Further testing showed that the lamellar phase is dominant even in

presence of impurities, as is illustrated in Figure SI2, which shows four distinct extents of the $DES \rightarrow IL$ reaction, with water and HexA remaining as excess reagents.

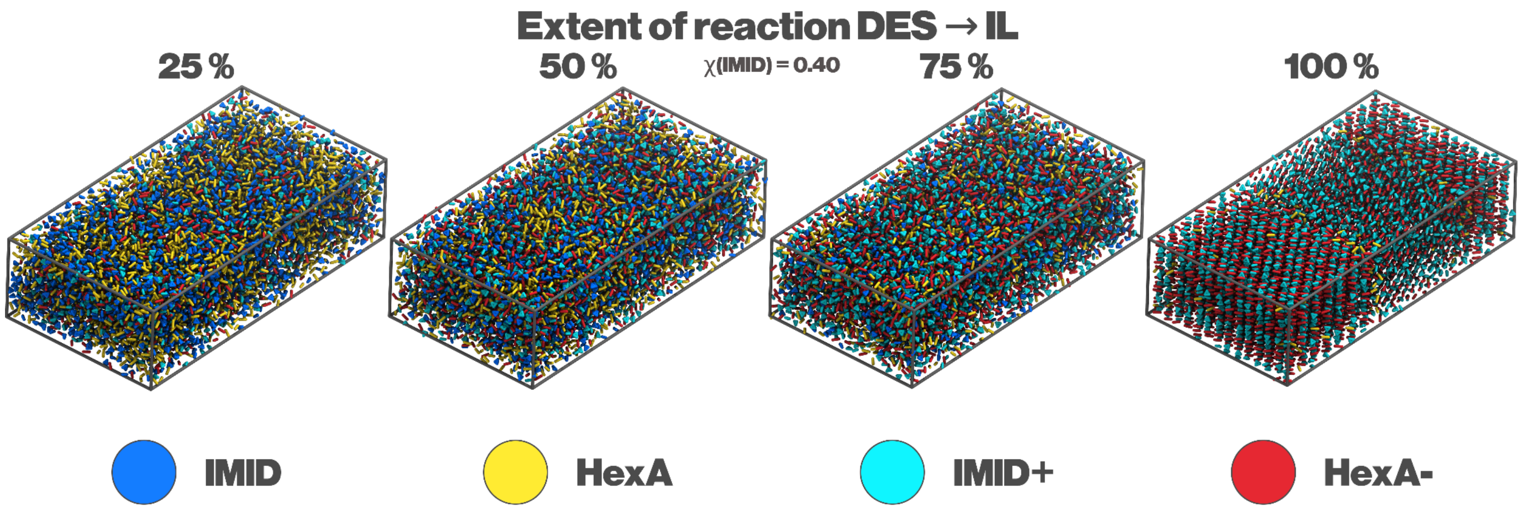

Figure S2: DES →IL reaction, with varying extents of reaction. Note the formation of a lamellar phase, even in the presence of water and excess HexA.

Further investigation of the fully reacted system indicated excess HexA preferentially residing at an interface between lamellar domains, and after aforementioned interfaces are saturated, HexA mixes in with the IL instead of forming a separate phase. Further testing with larger system sizes, such as the system depicted in Figure SI3, revealed this behaviour to be size-independent.

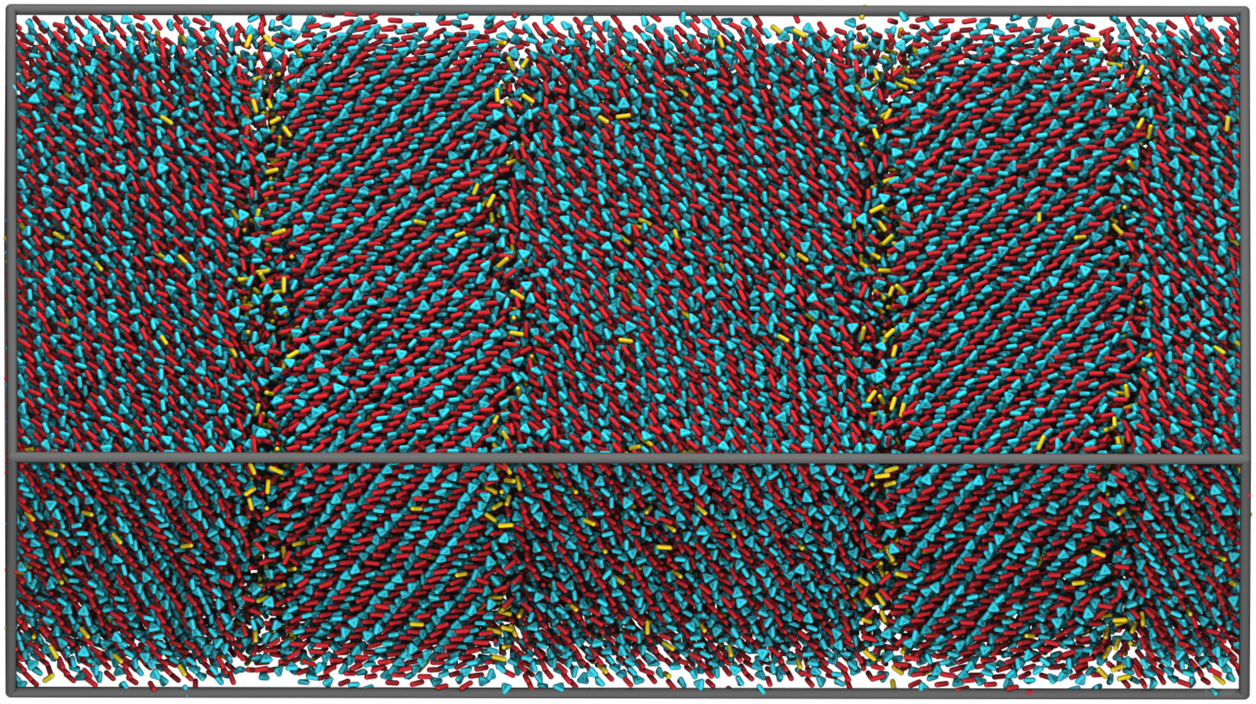

Figure S3: HexA (yellow) primarily residing at the IL interface. The colouring scheme is the same as in Figure S2.

Finally, we computed the density, molar volume, and viscosity at room temperature, and compared these values to those reported by Anouti *et al.*[6] Results of this comparison are given in Table SI3 below.

Table S3: Comparison of the Martini 3 [IMID+][HexA-] model against experimental values.

Property	This work	Anouti *et al.*
**Density (kg/m³)**	1031.05 ± 0.16	1024.6 ± 1.03
**Molar volume (cm³/mol)**	178.66 ± 0.02	179.7 ± 1.79
**Viscosity (mPa·s)**	363.05 ± 12.5	71.8 ± 0.07

3. Analysis of the number of contacts between IMID and HexA

Table S4: Percentage of the number of contacts between IMID and HexA.

χ(IMID)	270 K	280 K	290 K	300 K	310 K	320 K	330 K
**0.10**	63.7 %	63.8 %	64.0 %	64.3 %	64.3 %	64.3 %	64.4 %
**0.20**	50.9 %	51.4 %	51.8 %	52.2 %	52.5 %	52.7 %	52.9 %

**0.25**	44.6 %	45.0 %	46.2 %	46.6 %	47.2 %	47.4 %	47.5 %
**0.30**	39.3 %	40.4 %	41.0 %	41.5 %	42.0 %	42.2 %	42.5 %
**0.35**	34.0 %	35.3 %	36.1 %	36.7 %	37.4 %	37.6 %	38.0 %
**0.40**	28.8 %	30.5 %	31.5 %	31.9 %	33.0 %	33.2 %	33.8 %
**0.50**	20.7 %	22.6 %	23.6 %	24.3 %	24.9 %	25.6 %	25.9 %

4. Analysis of the number of contacts between IL and rest of the components

Table SI5 shows a similar analysis as was performed for the DES, but as a function of the extent of reaction. Temperature was kept constant at 310 K. The HBD:HBA ratio is still given as a mole fraction of imidazole (IMID). The extent of reaction indicates the proportion of IMID that was converted to IMID+, along with a corresponding amount of HexA being converted to HexA-. At χ(IMID) = 0.50 and above, $\xi = 100\,\%$ is omitted as $N(IMID) > N(HexA)$.

Table S5: Percentage of the number of contacts between the ionic liquid and other chemical species present in the system. The other components are water and HexA or water and IMID, depending on the extent of the reaction.

	**Extent of reaction** (ξ)			
**χ(IMID)**	**25 %**	**50 %**	**75 %**	**100 %**
**0.30**	59.7 %	45.9 %	32.6 %	19.0 %
**0.35**	60.0 %	44.0 %	27.4 %	10.5 %
**0.40**	60.2 %	42.8 %	23.9 %	3.2 %
**0.45**	52.4 %	41.4 %	19.9 %	7.2 %

**0.50**	60.3 %	39.7 %	21.0 %	-
**0.55**	60.3 %	37.9 %	31.6 %	-
**0.60**	60.2 %	36.8 %	36.4 %	-

The models predict a strong phase separation at low χ(IMID) when the reaction is allowed to proceed to completion. Interestingly, at lower extents of reaction, the system remains in a mixed state, suggesting that the formed IL can readily solubilize IMID. Furthermore, at $\xi = 75\ \%$ we observe the system moving from a partially mixed state to an almost biphasic state as the amount of IMID is increased, with even further addition of IMID reverting the system back to a partially mixed state. This is in line with the findings reported in Figure 8 in the main text, where only a weak phase separation is observed at $\xi = 100\ \%$.

5. Radial distribution functions

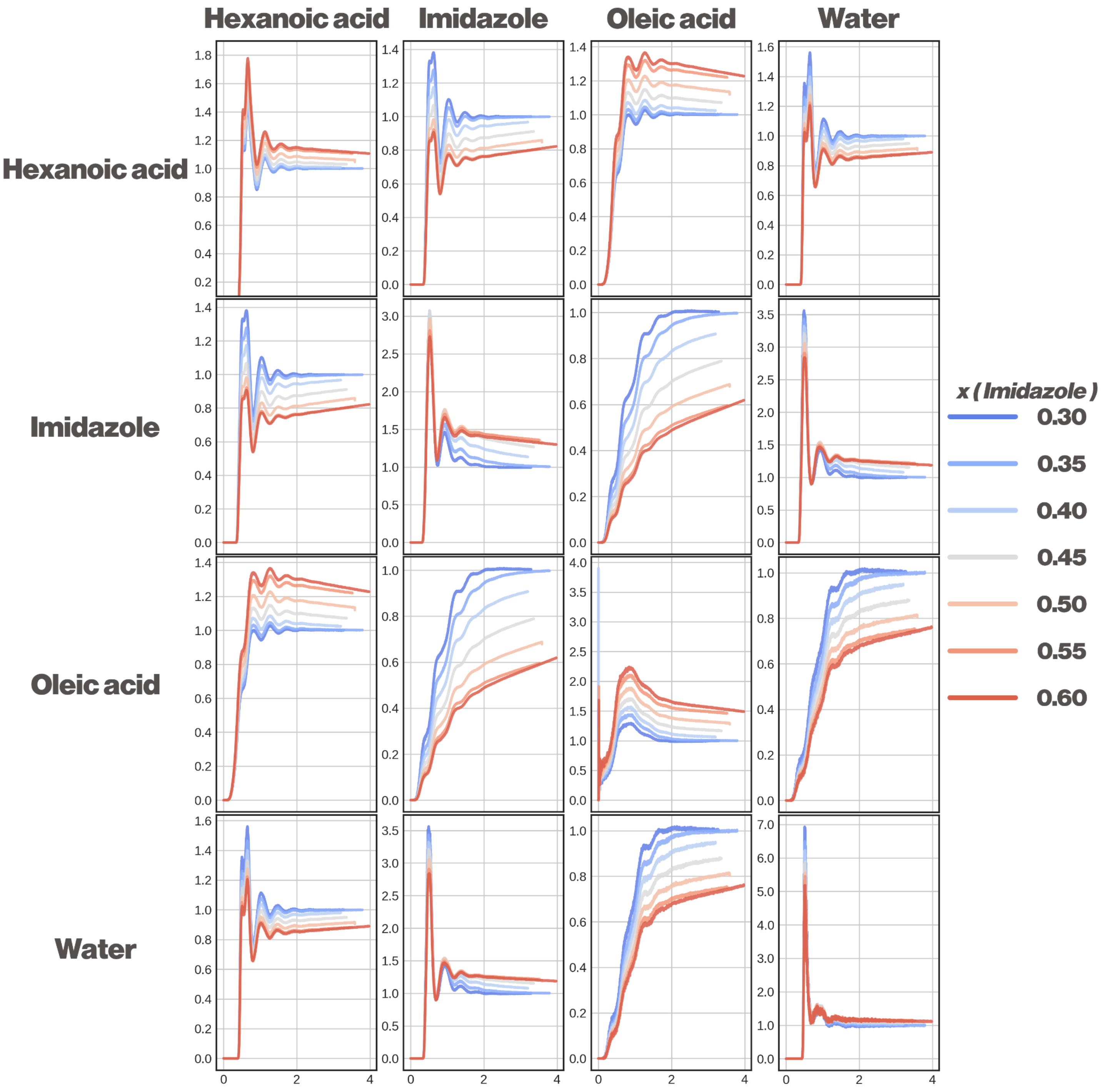

Figure S4: Radial distribution functions of the oleic acid containing systems, as a function of χ_{IMID}.

Table S6: Per-phase, finite-size corrected diffusion coefficients for Hexanoic Acid (HexA) and Imidazole (IMID) at various compositions and temperatures. The phase environment is defined by the local molecular composition: 'H-rich' refers to the HexA-rich phase and 'I-rich' refers to the IMID-rich phase. ‘nan‘ indicates that no molecules were found to originate in that phase, confirming the absence of stable phase separation under those conditions.

**Per-phase diffusion coefficients (10^{-5} cm^2/s)**				
**χ(IMID)**	**270 K**			
	**HexA (H-rich)**	**HexA (I-rich)**	**IMID (H-rich)**	**IMID (I-rich)**
**0.10**	1.875 ± 0.015	*nan*	2.573 ± 0.014	*nan*
**0.30**	1.868 ± 0.021	1.851 ± 0.017	2.420 ± 0.015	2.411 ± 0.031
**0.50**	1.648 ± 0.04	1.728 ± 0.015	2.140 ± 0.018	2.052 ± 0.014
	**300 K**			
	**HexA (H-rich)**	**HexA (I-rich)**	**IMID (H-rich)**	**IMID (I-rich)**
**0.10**	2.795 ± 0.024	*nan*	3.728 ± 0.020	*nan*
**0.30**	2.756 ± 0.022	2.796 ± 0.032	3.710 ± 0.052	3.609 ± 0.033
**0.50**	2.658 ± 0.022	2.701 ± 0.031	3.323 ± 0.019	3.371 ± 0.021
	**330 K**			
	**HexA (H-rich)**	**HexA (I-rich)**	**IMID (H-rich)**	**IMID (I-rich)**
**0.10**	3.918 ± 0.027	*nan*	5.407 ± 0.048	*nan*
**0.30**	3.825 ± 0.025	3.794 ± 0.024	5.023 ± 0.036	4.994 ± 0.037
**0.50**	3.793 ± 0.034	3.807 ± 0.038	4.764 ± 0.034	4.819 ± 0.048

References

1. Vainikka, P.; Thallmair, S.; Souza, P. C. T.; Marrink, S. J. Martini 3 Coarse-Grained Model for Type III Deep Eutectic Solvents: Thermodynamic, Structural, and Extraction Properties. *ACS Sust. Chem. & Eng*. **2021**, 9, 17338–17350.
2. Rowland, R. S.; Taylor, R. Intermolecular Nonbonded Contact Distances in Organic Crystal Structures: Comparison with Distances Expected from van der Waals Radii. *J. Phys. Chem.* **1996**, 100, 7384– 7391.
3. Hansch, C.; Dunn, W. J. Linear Relationships between Lipophilic Character and Biological Activity of Drugs. *J. Pharm. Sci*. **1972**, 61, 1– 19.
4. Sangster, J. Octanol‐Water Partition Coefficients of Simple Organic Compounds. *J. Phys.Chem*. Ref. Data. **1989**, 18, 1111–1229.
5. Vazquez-Salazar, L. I.; Selle, M.; de Vries, A. H.; Marrink, S. J.; Souza, P. C. T. Martini coarse-grained models of imidazolium-based ionic liquids: from nanostructural organization to liquid–liquid extraction. *Green Chem*. **2020**, 22, 7376–7386.
6. Anouti, M.; Jones, J.; Boisset, A.; Jacquemin, J.; Caillon-Cavanier, M.; Lemordant, D.; Aggregation behavior in water of new imidazolium and pyrrolidinium alkycarboxylates protic ionid liquids. *J. Colloid Interface Sci.* **2009**, 340, 104-111.
